# Inhibition of Autophagy and the Cytoprotective Role of Smac Mimetic against ROS-Induced Cancer: A Potential Therapeutic Strategy in Relapse and Chemoresistance Cases in Breast Cancer

**DOI:** 10.3390/cimb45070363

**Published:** 2023-07-10

**Authors:** Sahar Rafat, Mohammed Ageeli Hakami, Ali Hazazi, Ahad Amer Alsaiari, Summya Rashid, Mohammad Raghibul Hasan, Abdulaziz A. Aloliqi, Alaa Abdulaziz Eisa, Mohammad Irfan Dar, Mohd Faisal Khan, Kapil Dev

**Affiliations:** 1 Department of Biotechnology, Jamia Millia Islamia, New Delhi 110025, India; saharrafat18@gmail.com (S.R.);; 2 Department of Clinical Laboratory Sciences, College of Applied Medical Sciences, Shaqra University, Al-Quwayiyah, Riyadh 11911, Saudi Arabia; 3 Department of Pathology and Laboratory Medicine, Security Forces Hospital Program, Riyadh 11481, Saudi Arabia; 4 Department of Clinical Laboratory Sciences, College of Applied Medical Sciences, Taif University, Taif 21944, Saudi Arabia; 5 Department of Pharmacology & Toxicology, College of Pharmacy, Prince Sattam Bin Abdulaziz University, Al Kharj 11942, Saudi Arabia; 6 Department of Medical Biotechnology, College of Applied Medical Sciences, Qassim University, Buraydah 51542, Saudi Arabia; 7 Department of Medical Laboratories Technology, College of Applied Medical Sciences, Taibah University, Medina 42353, Saudi Arabia

**Keywords:** autophagy, apoptosis, ROS, Smac mimetics, cancer

## Abstract

With more than a million deaths each year, breast cancer is the top cause of death in women. Around 70% of breast cancers are hormonally responsive. Although several therapeutic options exist, cancer resistance and recurrence render them inefficient and insufficient. The major key reason behind this is the failure in the regulation of the cell death mechanism. In addition, ROS was also found to play a major role in this problem. The therapeutic benefits of Smac mimetic compound (SMC) BV6 on MCF7 were examined in the current study. Treatment with BV6 reduces viability and induces apoptosis in MCF7 breast cancer cells. BV6 suppresses autophagy and has demonstrated a defensive role in cancer cells against oxidative stress caused by H_2_O_2_. Overall, the present investigation shows that SMC has therapeutic and cytoprotective potential against oxidative stress in cancer cells. These Smac mimetic compounds may be used as anti-cancer drugs as well as antioxidants alone or in conjunction with other commonly used antioxidants.

## 1. Introduction

Changes in the expression, behaviors, and activities of encoded products of the genes brought on by various genetic and epigenetic mutations, environmental changes, and environmental variations are the main contributors to the variety of cancers that develop. The leading cause of cancer-related death among women is breast cancer (BC), with more than 24.5% of cancer incidence rates and 15.5% of cancer mortality rates, respectively [1]. Surgery, along with therapies like radiation therapy, chemotherapy, targeted therapy, and hormonal treatments, are all available options. Although there are treatments present, nonspecific targeting, cancer recurrence, resistance, and side effects render them inefficient and raise the mortality rate [2].

The most common type (approximately 70%) of breast cancer among women is hormone receptor-positive cells, i.e., estrogen (ER) and progesterone (PR) receptor-positive cells, which utilize body hormones for growth and proliferation. This breast cancer subtype is managed by surgery, radiation therapies, lowering body hormone levels, or drugs, such as tamoxifen, toremifene, and fulvestrant, to degrade or block the ER and PR receptors on cancer cells attaching to the ER and PR hormones. Despite having clinical benefits, many studies have demonstrated the reoccurrence of cancer and the development of resistance to these Selective estrogen receptor modulators (SERMs) and Selective estrogen receptor degraders (SERDs) drugs, along with various side effects. Many groups of researchers found both in vitro and in vivo that MCF7 cells (a study model of estrogen response) develop resistance to anti-estrogens and contribute to the progression of breast cancer [3,4,5,6,7]. The primary cause of unbridled cell growth is the absence or malfunction of mechanisms for programmed cell death (PCD), such as apoptosis and autophagy. Moreover, failure in the regulation or any alteration, overexpression, or under expression of the proteins of these PCD mechanisms is one of the key reasons [8,9].

Apoptosis, also referred to as type I cell death, is a tightly regulated PCD. Inhibitors of Apoptosis proteins (IAPs) are a group of proteins found to prevent cell death by inhibiting caspase activation. IAPs bind to caspases through Baculoviral IAP repat (BIR) domains. A small endogenous protein called the second mitochondrial-derived activator of caspases (Smac) is released into the cytoplasm from the mitochondria upon apoptosis activation. The Smac protein releases the caspases (IAPs bound) and binds to the inhibitors of apoptosis (IAPs) proteins. Smac proteins inhibit the action of IAPs and have been found to be downregulated in cancer. Cancer recurrence and resistance to anticancer therapy are also strongly found to be correlated with anti-apoptotic Bcl2 family proteins, including Bcl2, Bcl-xL, Bcl-w, Mcl-1, A1/BFL-1, etc. [10]. In addition to apoptosis, autophagy is another highly regulated, stress-adaptive recycling mechanism that includes the lysosomal system. It helps maintain homeostasis and supplies nutrition. In brief, the cellular components or unwanted or misfolded proteins/enzymes that have to be degraded are brought by cargo proteins, such as p62/sequestome1, to the elongating phagophore and get encapsulated into the double membrane autophagy vesicles known as autophagosomes. Furthermore, mature substrate encapsulated autophagosomes fuse with lysosomes to form autolysosomes. The acidic components of lysosomes and enzymes break down and degrade the substrates into simpler substances. The waste is removed via exocytosis, and the simple building blocks of fats, proteins, and carbohydrates are supplied back to the cell [11]. Various diseases may develop as a consequence of process failure. Autophagy has a dual role in cancer, inhibiting the onset of tumors (acting as a tumor suppressor) and also promoting tumor growth [11].

Autophagy is a fundamental mechanism for cellular homeostasis, especially in the context of oxidative stress. Overproduction of ROS, which is the root cause of oxidative stress, is brought on by endogenous and external sources as well as ineffective antioxidant mechanisms. Similar to autophagy, ROS has a dual role in carcinogenesis [12,13]. Different therapies trigger ROS-induced autophagy, which either results in the development of drug resistance or the initiation of apoptosis [14,15,16].

**Figure 1:**
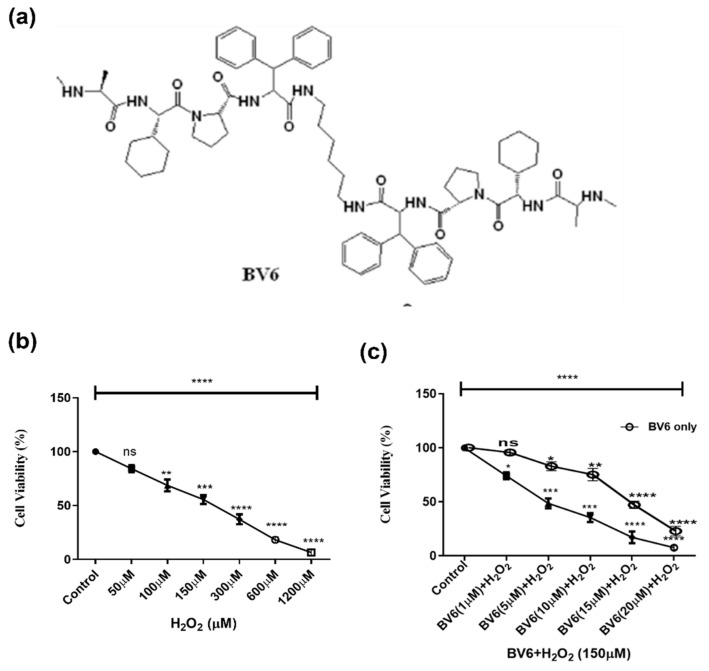
(**a**) The chemical structure of BV6 [23]. (**b**) Effect of an increasing concentration of H_2_O_2_ (0–1200 µM) and (**c**) BV6 with H_2_O_2_ (100 µM) on the viability of cancer cells measured by MTT assay. Cells were treated for 24 h. MTT incubation for 3 h, followed by DMSO, and the results were measured at a wavelength of 570 nm. The experiment was performed in triplicate, and data from three different trials are presented as mean ± SD. (*), (**), (***), and (****) show *p* ˂ 0.05, *p* ˂ 0.01, *p* ˂ 0.001, and *p* ˂ 0.0001, respectively, for each case.

Evidence exists that supports Smac mimetics suppressing IAP expression. In addition to assisting with apoptosis, it also makes resistant cancer cells receptive to medicines. In pre-clinical and clinical investigations, different Smac mimetic compounds (SMC) are demonstrating their potential against cancer [17,18,19,20]. In the current study, we explore the therapeutic potential of BV6 [N,N′-(hexane-1,6-diyl) bis (1 {(2S)-2-cyclohexyl-2-[(N-methyl-L-alanyl)-amino]-acetyl}-L-prolyl-beta-phenyl-L phenylalaninamide)] (Figure 1a), a Smac mimic on H_2_O_2_-induced oxidatively stressed MCF7 breast cancer cells. We investigated cell viability and apoptosis. We also examine its impact on H_2_O_2_-induced oxidative stress and autophagy.

## 2. Material and Methods

### 2.1. Cancer Cell Culture

Under sterile conditions, MCF-7 purchased from the National Centre for Cell Science (NCCS), Pune, India, were grown and maintained at 37 °C in a humidified environment with 5% CO_2_ in Dulbecco’s modified Eagle’s medium (DMEM) enriched with 10% (*vol*/*vol*) heat-inactivated FBS (Gibco, São Paulo, Brazil) and 1% penicillin/streptomycin (Himedia, Mumbai, India). After 3–4 days, the cultured cells were routinely split/passed and placed into a fresh cell culture flask. To Split or detach cells from the surface for experiments, pre-warmed (37 °C) 0.05% Trypsin (Gibco) was added to the cell monolayer and suspended in four times the volume of complete growth media by gentle pipetting.

### 2.2. MTT Assay

To assess the viability of cells for compound BV6 (Calbiochem, San Diego, CA, USA), with and without hydrogen peroxide (H_2_O_2_), cells were grown and treated with different concentrations of BV6 (1 µM, 5 µM, 10 µM, 15 µM, and 20 µM) and H_2_O_2_ (50 µM, 100 µM, 150 µM, 300 µM, 600 µM, and 1200 µM) alone and in combination for 24 h in 96-well microtiter plates (8000 cells/well). MTT (20 µL per well) was added and incubated at 37 °C for 3 h. In order to dissolve the formazan crystals formed, 100 µL of DMSO was given to each well after the media containing MTT (Himedia, Mumbai, India) was aspirated. After one hour, at a wavelength of 570 nm and a reference wavelength of 630 nm, the plate was examined using a microtiter plate reader (Bio-Rad, Hercules, CA, USA). The formula used to determine cell viability (%) was given as follows:

$$\mathrm{cell}\;\mathrm{viability}\;(\%)=\frac{\mathrm{absorbance}\;\mathrm{of}\;\mathrm{test}\;\mathrm{sample}}{\mathrm{absorbance}\;\mathrm{of}\;\mathrm{control}}\times 100$$

### 2.3. DAPI Staining

MCF-7 cells (0.5 × 10^5^) were cultured on coverslips (50% confluency) and treated with BV6 and H_2_O_2_ for 24 h. After fixing with ice-cold Methanol for 10 min, cells stained with 1 μg/mL DAPI (406-diamidino-2-phenylindole) in PBS were incubated and then rinsed with PBS for 5 min each. Cells were then observed under a confocal microscope at 350 nm of excitation wavelength.

### 2.4. AO/EtBr (Acridine Orange and Ethidium Bromide) Staining

Live, apoptotic, and necrotic cells can all be stained using acridine orange and ethidium bromide. Cells (1 × 10^5^) plated in a 12-well plate that had reached more than 80% confluency were treated. After being trypsinized, centrifuged (1000 rpm), and cleaned with PBS, cells were resuspended in an AO/EtBr solution (1:1). After 15 min of incubation with AO/EtBr, cells were examined under a fluorescence microscope by placing a drop of cell suspension on a clean glass slide.

### 2.5. Flow Cytometry

Using Annexin V/Propidium Iodide (PI) (BD Biosciences, San Jose, CA, USA) and DCFDA staining, the apoptosis and ROS status of cells after treatment with BV6 and H_2_O_2_ were evaluated. The cells were stained in accordance with the manufacturer’s instructions after 24 h of treatment. Using flow cytometry, the fluorescence intensity was evaluated (BD Aria, Lenoir, NC, USA).

### 2.6. MDC (Monodansylcadavarine) Assay

The cells were cultured on coverslips for 24 h (about 50% confluent) before being exposed to BV6 and H_2_O_2_ in a growth medium for a further 24 h. After 5 min of rinsing with phosphate-buffered solution, the cells were stained with MDC (Monodansylcadavarine) dye for 30 min at 37 degrees. This was followed by 10 min of 40 mM NH_4_Cl incubation to lessen the staining of lysosomes. After a gentle PBS wash, a cover slip with attached cells was placed on the slide and examined under a fluorescence microscope. In addition, a 96-well plate was also prepared following the same procedure to measure the fluorescence intensity of the dye using a fluorescence microtiter plate reader [21].

### 2.7. Cytoprotective Impact Measured against ROS-Induced Cellular Oxidative Stress

In accordance with the manufacturer’s recommendations, measurement of ROS was carried out using 2′,7′-dichlorofluorescin diacetate (DCFDA), a fluorescein dye. After administering the drug, the media was taken out and 100 μM H_2_O_2_ was added for an hour to elicit oxidative stress in the cell lines. Dilute 10 mM DCFDA (Sigma Aldrich, St. Louis, MO, USA) in a clear medium and treat at 37 °C for 30 min. Under a fluorescent microscope, cells were photographed after being washed three times with clear medium. A flow cytometer (BD, Aria, Lenoir, NC, USA) was also utilized to detect the DCF fluorescence at excitation 488 nm and emission 525 nm, respectively. In addition, we incubated the cells for 2 h with ascorbic acid, a strong antioxidant [22].

### 2.8. Real-Time PCR

RNA (total) was isolated using TRIzol Reagent (Invitrogen, Waltham, MA, USA). By using the manufacturer’s suggested guidelines for the Verso cDNA Synthesis Kit (Thermo Scientific, Vilnius, Lithuania), 1 μg of RNA was reverse transcribed to form cDNA. Real-time PCR was performed with SYBR green technology on an Applied Biosystems (Foster City, CA, USA) real-time PCR machine. Autophagy biomarkers, such as Beclin1 and LC3 primers, were synthesized by IDT (Coralville, IA, USA) and are as follows: Pair A. Beclin-1: Sense 5′-AGG ATG ATG TCC ACA GAA AGT GC-3′Anti-sense 5′-AGT GAC CTT CAG TCT TCG GCT G-3′ Pair B. LC-3: Sense 5′-AGA CCT TCA AGC AGC GCC G-3′Anti-sense 5′-ACA CTG ACA ATT TCA TCC CG-3′ Pair G. Actin Primers: Sense 5′-CCC CTT CAT TGA CCT CAA CT-3′ Antisense 5′-TTG TCA TGG ATG ACC TTG GC-3′.

### 2.9. Western Blotting

Following treatment, the protein was extracted using RIPA lysis buffer and protease inhibitor (Promega, Madison, WI, USA), which was followed by incubation on ice and centrifugation (16,000 rpm) for 20 min each. The supernatant was collected in a fresh tube, and the concentration of protein was assessed using the Bradford assay. After the SDS-PAGE gel (12%) resolution of the 30–50 µg of total protein, Western blotting was performed to transfer protein from the gel to the PVDF membrane (Bio-Rad, Hercules, CA, USA) at 75 V for 30 to 2 h. 5% nonfat milk for an hour was used to block the membrane, followed by overnight 4 °C probing with the primary antibodies MAP LC3a/b (sc-398822), Beclin 1 (sc-48341), and a housekeeping gene-specific antibody, i.e., Actin (sc-47778, Santa Cruz Biotechnology, Dallas, TX, USA). The unbound antibodies were removed, followed by HRP-conjugated secondary antibodies (sc-516102-CM, Santa Cruz Biotechnology) incubation for 1 h. The membrane was overlaid with a chemiluminescence-based ECL substrate from Bio-rad (USA), and signals were seen and recorded using Chemidoc (Bio-Rad, Hercules, CA, USA).

### 2.10. Transmission Electron Microscope (TEM)

Cancer cells, both untreated and treated, were collected and fixed in Karnovsky’s fixative solution (3% glutaraldehyde) at 4 °C overnight. The samples were implanted in an epoxy resin after postfixation with 1% osmic acid and step-wise gradient ethanol dehydration (30–100%). Samples were collected on copper grids after being cut into incredibly small slices with an ultramicrotome. The electron microscope was used to inspect grids (CM-10, Philips, Cambridge, MA, USA).

### 2.11. Statistical Analysis

The data was analyzed using statistical software, Graph Pad Prism version 8.0. The results are shown as the standard deviation (SD) from three independent experiments. Statistical significance was determined by Tukey’s multiple comparison tests and the ANOVA at *p* < 0.05. The p values less than *p* < 0.05, *p* < 0.01, *p* < 0.001, and *p* < 0.0001 are denoted by the asterisks (*), (**), (***), and (****), respectively.

## 3. Results

### 3.1. Effect of Smac Mimetic Compound BV6 with H_2_O_2_ on Cell Viability

MCF-7 breast cancer cells were treated with different concentrations of H_2_O_2_ to find an optimal concentration to induce oxidative stress with low cellular loss. The concentration of 100 μM of H_2_O_2_ was used to cause oxidation since there was less significant loss of cell viability (Figure 1b). Cells after 24 h of incubation with H_2_O_2_ and BV6 in the presence of H_2_O_2_ displayed a decrease in cell viability with an increase in concentration. About 70% and 50% reductions in cell viability were found at 1 μM and 5 μM of BV6 with H_2_O_2_, and 10 μM and 15 μM of BV6 without H_2_O_2,_ respectively (Figure 1c). According to our findings, after 24 h of treatment, cell viability is gradually dose-dependently inhibited. Statistics support the significance of the data presented.

### 3.2. BV6 Effect on ROS in H_2_O_2_-Induced Breast Cancer Cells

**Figure 2:**
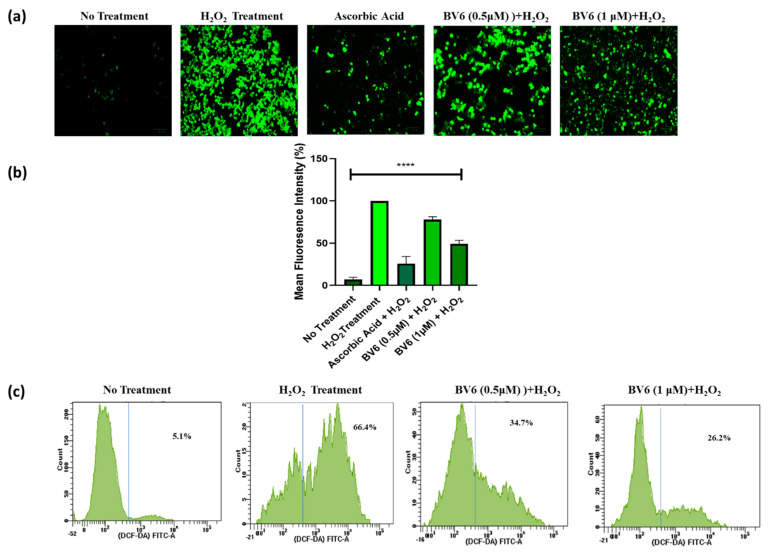
Evaluation of BV6 cytoprotective activity in cells exposed to H_2_O_2_: Cells were rinsed and exposed to 100 mM H_2_O_2_ for 45 min after receiving BV6 treatment for 24 h and dyed for 30 min with DCFDA. Cells were taken and examined under a fluorescent microscope (**a**). Cells in a representative image with fluorescence distribution; scale bar = 100 µm. (**b**) The mean fluorescence intensity of H_2_O_2_ and BV6-treated cells. (**c**) Flow cytometry analysis of ROS induced by H_2_O_2_ in MCF7 cells treated with BV6. (****) show *p* < 0.0001, respectively, and are carried out in triplicate and displayed as mean ± SD.

Intracellular oxidative stress, i.e., ROS levels, was investigated by FACS and microscopy using a fluorescent probe, DCFH-DA, which is nonpolar and nonionic and passively diffuses into the cell [24]. In addition to optimum concentration, DCF had high fluorescence intensity at 100 μM of H_2_O_2_ [25]. The cells were pre-treated with BV6 for 24 h and exposed to hydrogen peroxide (H_2_O_2_). The DCFDA fluorescent images and fluorescence intensity show that treatment with H_2_O_2_ increases ROS production, and cells pre-treated with BV6 and ascorbic acid significantly reduce H_2_O_2_-induced oxidative stress/ROS. Compared to the H_2_O_2_ treatment only having a high fluorescence intensity (100%), ascorbic acid reduces the intensity (ROS) by 75%, whereas BV6 reduces the ROS by 25% to 50% in a dose-dependent manner (Figure 2a,b). Flow cytometry analysis revealed that BV6 reduces 32% to 40% of ROS in a dose-dependent manner compared to H_2_O_2_ treatment, which only reduced 66.4% of ROS (Figure 2c). This demonstrates that Smac mimetic compounds provide, at least in part, an antioxidant capacity to defend cells from the problem of oxidative stress. The protective effect of BV6 against H_2_O_2_-induced oxidative stress in breast cancer cells is studied.

### 3.3. Combinational Treatment of BV6 with H_2_O_2_ (100 µM) Enhances Apoptosis in MCF-7 Cells

**Figure 3:**
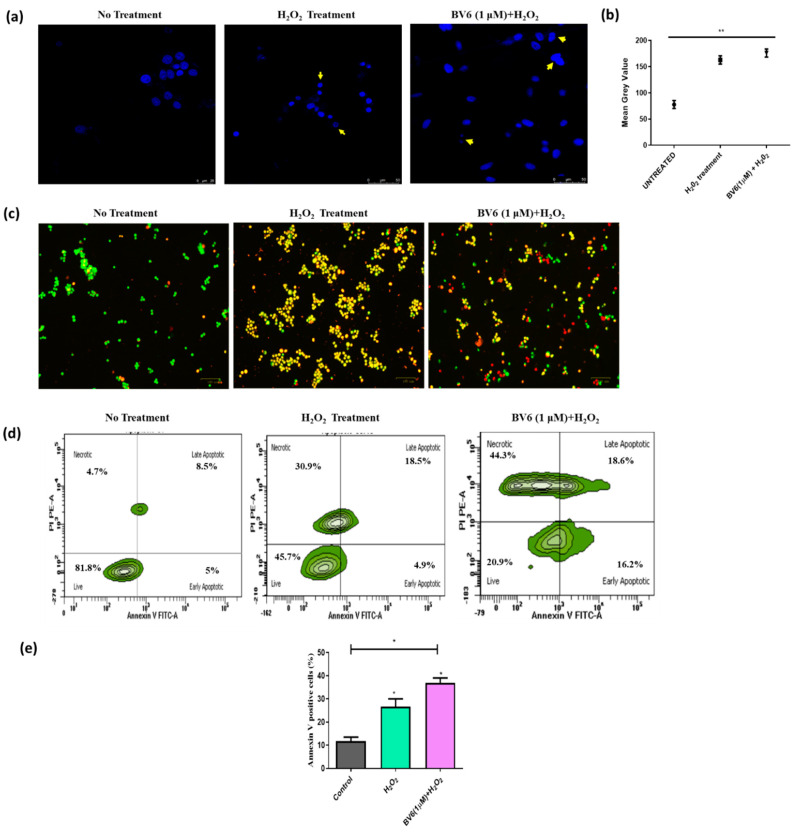
Apoptosis analysis: Treatment of BV6 with H_2_O_2_ (100 µM) for 24 h shows enhanced apoptosis induction compared to treatment with H_2_O_2_ (100 µM). (**a**) The confocal microscopy images show cells stained with DAPI. Yellow arrows demonstrate nuclear morphology changes in response to H_2_O_2_ (100 µM) with and without the Smac mimetic compound BV6 (1 µM). Scale bar = 50 µm. (**b**) The dot plot shows the cells mean gray value to quantify the DAPI fluorescence signals. Scale bar = 100 µm. (**c**) Images showing cells stained with AO/EtBr after treatment. (**d**) The quadrants after flow cytometry using Annexin V/PI stain display the live cells, early apoptotic cells, late apoptotic cells, and necrotic cells proportion. (**e**) Graphical representation of Annexin V-positive cells (%) in response to treatment. The data from three different trials is presented as mean ± SD and is statistically significant. (*), (**) show *p* < 0.05, *p* < 0.01, respectively, for each case.

Apoptosis and cell death were studied in BV6-treated MCF7 cells with and without H_2_O_2_ (100 µM) by DAPI staining to observe nuclear changes, AO/EtBr staining, and Annexin V/PI staining to observe early apoptosis, late apoptosis, and necrosis. After 24 h treatment of H_2_O_2_ (100 µM) and BV6 with H_2_O_2_ (100 µM), changes in the nuclear morphology were observed, like apoptotic body formation, blebbing, chromatin condensation, and fragmentation (yellow arrows in confocal images) (Figure 3a,b). Whereas control cells displayed an intact nucleus. In Figure 3c, fluorescence images of cells stained with AO/EtBr, exemplifying green, orange, yellow, and red fluorescence, respectively, represent living cells, early apoptotic cells, late apoptotic cells, and necrotic cells. Additionally, flow cytometry of Annexin V/PI indicates cell groups that are going through apoptosis. Early apoptotic cells are AnnexinV+/PI− whereas late apoptotic cells are AnnexinV+/PI+. While AnnexinV−/PI− stained cells are alive, AnnexinV−/PI+ stained cells are necrotic cells. (Figure 3d). BV6 treated with H_2_O_2_ (100 µM) shows an approximately 15% increase in annexin-positive cells compared to the H_2_O_2_-treated (100 µM) annexin-positive cells (Figure 3d,e). In addition, a 25–40% increase in the necrotic cell population was observed after treatment with H_2_O_2_ (100 µM) and BV6 with H_2_O_2_, respectively.

### 3.4. Autophagy Is Downregulated by the BV6 Compound in H_2_O_2_-Induced Breast Cancer Cells

**Figure 4:**
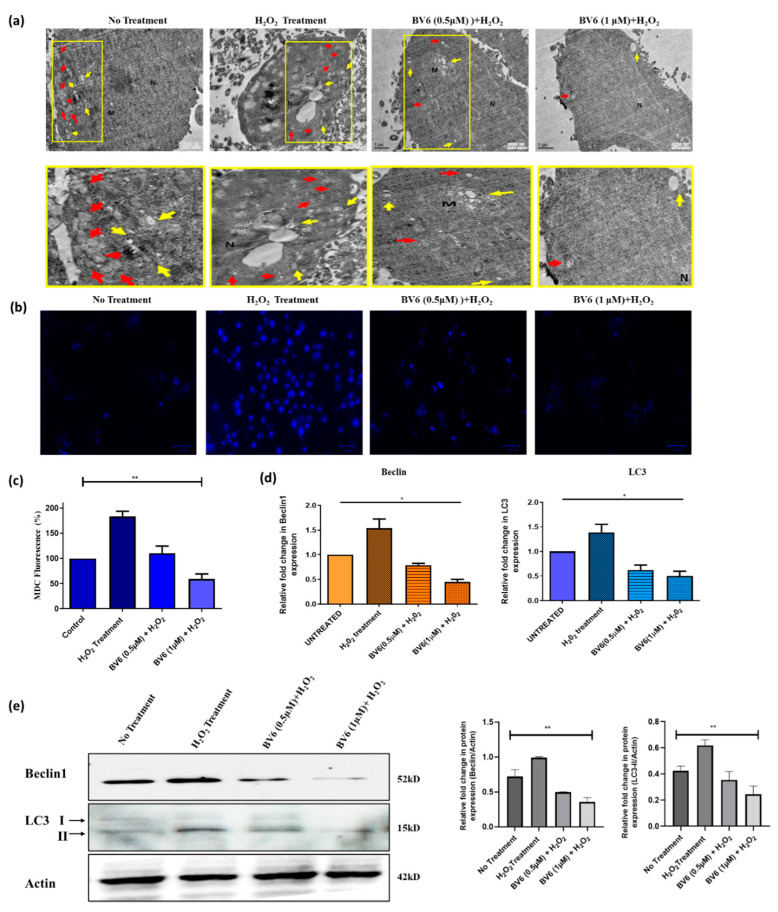
Detection of autophagy in H_2_O_2_-induced breast cancer cells: (**a**) Transmission electron microscopy images displaying autophagic vacuoles in red arrows (autophagosomes) and yellow arrows (autolysosomes). M and N are mitochondria and nuclei, respectively. Scale bar = 1 µm. Images with yellow highlighted borders are the zoom images of the above TEM pictures. (**b**) MDC dye fluorescence microscopic images demonstrating the accumulated autophagy vacuoles in response to H_2_O_2_ and BV6. Scale bar = 34 µm. (**c**) The relative change in the mean MDC dye fluorescence intensity is demonstrated by the histogram profile. (**d**) Fold change in Beclin1 and LC3 mRNA expression levels normalized by Actin after 24 h of treatment. (**e**) a Western blot demonstrating the protein expression of autophagic biomarkers, such as Beclin1 and LC3-II, in response to the treatment of BV6 and H_2_O_2._ Graphs represent the quantification of protein expression levels normalized by Actin. The data from three different trials is presented as mean ± SD and is statistically significant. (*) and (**) show *p* ˂ 0.05, *p* ˂ 0.01, respectively, for each case.

Autophagy was qualitatively evaluated by TEM, demonstrating a decrease in autophagic vacuoles, i.e., autophagosomes (Red arrows) and autolysosomes (yellow arrows). The TEM images suggest that autophagy decreases in response to BV6 (Figure 4a). Additionally, 50 mM MDC dye was used to color both H_2_O_2_-treated cells with the BV6 compound and untreated cells (control). MCF7 cell fluorescence images demonstrate autophagosomes. Quantifying fluorescence intensity gives an understanding of how autophagy is altered in BV6 and H_2_O_2_-treated cells compared to control groups. In the histogram profile (Figure 4b,c), H_2_O_2_-treated cells show high fluorescence (175%) following a decrease in fluorescence intensity with an increase in dose concentration of BV6. BV6 doses of 0.5 μM and 1.0 μM applied to H_2_O_2_-induced breast cancer cells show a 50% decrease in intensity, whereas there is a 100% decrease, respectively, compared to the H_2_O_2_-treated cells. Moreover, the protein as well as the mRNA expression of autophagy biomarkers, i.e., Beclin1 and LC3, is found to be downregulated in H_2_O_2_-induced cancer cells after 24 h of treatment with an increasing dose of BV6 (Figure 4d,e). Hence, the results suggest that autophagy is downregulated in response to BV6 in H_2_O_2_-induced breast cancer cell lines.

## 4. Discussion

The leading cause of mortality worldwide is cancer, approximately more than 13%, and chemotherapy and radiotherapy are the most frequently used. However, in hormone receptor (ER and PR)-positive cells, hormone therapy is the most commonly used and only option. These treatments come with a surfeit of side effects, especially the extreme production of reactive oxygen species (ROS) and the consequent buildup of oxidative stress. In various cellular processes, such as senescence, apoptosis, autophagy, etc., ROSs are acknowledged to play a crucial role in regulating protein expression and function [26,27]. ROS levels are slightly elevated during the early stages of cancer and as it progresses. As a result, ROS levels in cancer cells are somewhat higher than in normal cells. When ROS-generating drugs are used in anticancer therapy, oxidative stress supersedes inherent stress, which causes tumor cells to be more likely to die or have their growth slowed. [28]. During therapies, the antioxidant mechanism may not be able to prevent the ROS’s adverse effect on critical cellular processes [29]. When using this approach, these agents enhance cytotoxicity, which would affect cancer cells as well as normal cells. Integrated cell cycle and apoptotic processes are necessary for the optimal cytotoxic activity of anti-neoplastic agents [30]. Many drugs are used for cancer chemotherapy that cause oxidative stress, which can interfere with antineoplastic activity and the efficacy of the treatment.

Many dietary supplements have tried to reduce these undesirable side effects, but antioxidants have become more popular as chemotherapy additives. However, because these supplements may conflict with treatments that prevent cancer by producing free radicals, many oncologists recommend against using antioxidant-rich dietary supplements. Antioxidants like vitamin E, N-acetylcysteine, and others have been demonstrated in numerous recent studies to enhance tumor growth and metastasis [29,31,32,33,34]. In another study, administering a combination of carotenoids was often found to be associated with a high risk of death from breast cancer and overall mortality. [35]. To maintain cellular homeostasis, ROS and autophagy work in tight regulation. In cancer, both have paradoxical roles: they either aid cancer cells to adapt to stress or induce cell death. Recent research has demonstrated that ROS and the autophagic pathway intersect in a profound manner. ROS aids in triggering autophagy [32]. Together, the information demonstrates, in particular, how the impacts of ROS and autophagy vary depending on the stage at which the tumor is developing [36]. Since ROS and autophagy both serve dual and paradoxical roles in cancer, supplementing antioxidants or inhibitors of autophagy occasionally may also reduce ROS- and autophagy-induced resistance/reoccurrence, which may lead to the induction of cell death [13,14,16,37,38,39].

In our study, we treated breast cancer cells with H_2_O_2_ to model therapies induced oxidative stress and investigated the antioxidant, protective properties, and autophagy status of the Smac mimetic BV6 compound in response to the induced oxidative stress. Figure 1 shows that the BV6 compound causes cancer cell death in a dose-dependent manner under oxidative stress. Compared to our previous published data, BV6 at 1 µM causes 10% cell death alone compared to BV6 at 1 µM in induced oxidative stress, i.e., 30% [16,40,41]. In addition, the pro-apoptotic action and the anti-cancer properties of the compound were substantiated by the treatment’s appearance of tell-tale signs of apoptosis, such as a change in the morphology of the nucleus (apoptotic bodies, blebbing, and chromatin condensation), which was further confirmed by Annexin V/PI staining. The BV6 under oxidative stress displayed a higher number of cells undergoing apoptosis and necrosis in comparison to control and H_2_O_2_-treated cells (Figure 3). To the best of our knowledge, BV6’s influence on autophagy and the cytoprotective activity it exerts on breast cancer cell lines are being studied for the first time. Our study revealed that the Smac mimetic compound BV6, in a concentration-dependent manner, reduced the ROS in H_2_O_2_-induced breast cancer cells. A decline in DCF fluorescence intensity corroborates the BV6 cytoprotective effect against ROS (Figure 2). Furthermore, the TEM, MDC dye staining, real-time PCR, and Western blot findings support the idea that BV6 downregulates autophagy in the H_2_O_2_-induced oxidative MCF7 cancer cell lines (Figure 4). Autophagy is found as a survival mechanism in most cancers and a factor in the development of cancer resistance and recurrence. In support of our result, a study demonstrates that the Smac mimics LCL161 and TL32711 prevent the production of reactive oxygen species (ROS) when doxorubicin, etoposide, or the activation of a 4OHT-inducible FOXO3 allele are used as chemotherapeutics [42]. This intriguing discovery reveals a “dark side” of SMAC-mimetics in cancer therapy and may reflect why phase I and phase II trials on SMAC-mimetics have been unsuccessful in recent decades [(https://clinicaltrials.gov; NCT02147873) (accessed on 8 August 2021)].

**Figure 5:**
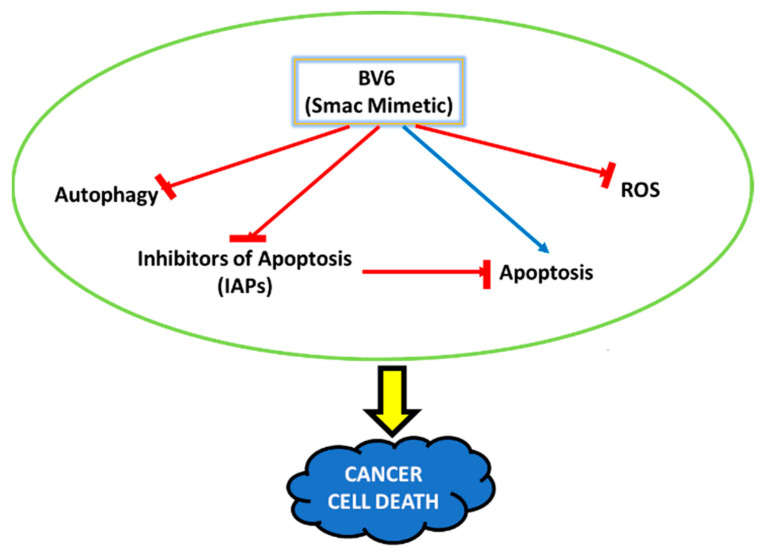
Schematic representation of the therapeutic potential and the changes in the cellular activities instigated by the Smac mimetic compound BV6. The compound identified to decrease autophagy, ROS, and inhibitors of apoptosis (IAPs). The Smac mimetic compound BV6 leads to cell death via the resumption of apoptosis in cancer cells.

Our present study and recently published studies conclude (Figure 5) that Smac mimetic BV6 decreases the factors causing cancer cell resistance against therapies, such as IAPs, autophagy, and ROS [16,40,41]. In order to safeguard against cancer relapse and resistance, SMC might be used either alone or in combination with standard anticancer therapy. To overcome the major obstacles of resistance and reoccurrence and the life-threatening side effects of the therapies in cancer patients, these Smac mimetic compounds might show a promising effect or could be used in place of or in amalgamation with other antioxidants during treatment. It is important to understand the cellular and molecular events happening in cancer to regulate and balance the ROS, autophagy, and apoptosis mechanisms.

## Data Availability

The data used to support the findings of this study are included within the article.
